# Microalgal Protein Extraction From *Chlorella vulgaris FSP-E* Using Triphasic Partitioning Technique With Sonication

**DOI:** 10.3389/fbioe.2019.00396

**Published:** 2019-12-06

**Authors:** Shir Reen Chia, Kit Wayne Chew, Hayyiratul Fatimah Mohd Zaid, Dinh-Toi Chu, Yang Tao, Pau Loke Show

**Affiliations:** ^1^Department of Chemical and Environmental Engineering, Faculty of Science and Engineering, University of Nottingham Malaysia, Semenyih, Malaysia; ^2^Faculty of Science and Engineering, School of Mathematical Sciences, University of Nottingham Malaysia, Semenyih, Malaysia; ^3^Fundamental and Applied Sciences Department, Centre of Innovative Nanostructures & Nanodevices (COINN), Institute of Autonomous System, Universiti Teknologi PETRONAS, Bandar Seri Iskandar, Malaysia; ^4^Faculty of Biology, Hanoi National University of Education, Hanoi, Vietnam; ^5^College of Food Science and Technology, Nanjing Agricultural University, Nanjing, China

**Keywords:** protein extraction, ultrasonic, triphasic partitioning technique, microalgae, bio-separation

## Abstract

Green microalgae containing various bioactive compounds and macronutrients such as lipids, carbohydrates, and proteins, have attracted much attention from the global community. Microalgae has the potential to be applied in food industries due to its high protein content, rapid growth rate, and ability to survive in harsh conditions. This study presents a simple yet efficient technique of sonication-assisted triphasic partitioning process, also known as ultrasonic-assisted three phase partitioning (UATPP), for the extraction of proteins from *Chlorella vulgaris* FSP-E. Comparison studies between three phase partitioning (TPP) and UATPP was conducted to investigate the feasibility of the enhanced technique on proteins extraction. Types of salt, ratio of slurry to t-butanol, salt saturation, sonication frequency, power, irradiation time, and duty cycle as well as biomass loading were studied. UATPP was found to be an improved technique compared to TPP. An optimum separation efficiency and yield of 74.59 ± 0.45 and 56.57 ± 3.70% was obtained, respectively, with the optimized conditions: salt saturation (50%), slurry to t-butanol ratio (1:2), sonication power (100%), irradiation time (10 min), frequency (35 kHz), duty cycle (80%) and biomass loading (0.75 wt%). A scaled-up study was performed to validate the reliability of UATPP for protein extraction. The outcome of the study revealed that UATPP is an attractive approach for downstream processing of microalgae.

## Introduction

Nowadays, the growth of global human population is increasing at an incredible speed each year. An estimated 70% increase in food production will be needed for the growing human population (by around 2.3 billion) by the year 2050 (Tester and Langridge, 2010). The remarkable supply growth of food productions have reduced the proportion of global hunger, despite the world population doubling over the last half-century (Bleakley and Hayes, 2017). Nevertheless, the world is facing a great challenge in sustaining adequate food production to meet the rising demands. Methods and techniques used conventionally to produce food will soon no longer be a solution due to the emission of greenhouse gases by these processes, nutrient run-off causing environmental pollutions, degradation of soil and disruption of ecosystem caused by over-harvesting of aquatic foods (Tester and Langridge, 2010). Specifically, protein is one of the macronutrients that will be in shortage at the near future. A substitution or alternative protein source and more efficient production techniques needs to be discovered and developed in order to meet the global demand.

Microalgae is well-known as a viable source of biological components such as carbohydrates, lipids, pigments, vitamins, and polyphenols, especially proteins (Hsieh and Wu, 2009; Chia et al., 2018). The protein quality in microalgae is known to be similar with some of the traditional protein sources like milk, meat, and egg (Bleakley and Hayes, 2017). In general, the protein content of microalgae constitutes a major portion compared to lipid and carbohydrate (Lavens and Sorgeloos, 1996). The total protein content from microalgae, especially *Chlorella* sp. is about 43–50% (Ramazanov and Ramazanov, 2006; Phukan et al., 2011). In the studies of Thompson et al. (1996) and Richmond (2017), the nutritive quality of *Chlorella sp*. was proved to be influenced by light environment during the microalgae cultivation stage (Thompson et al., 1996; Richmond, 2017). Higher protein production was obtained with the increment of light irradiance and longer photoperiod (Seyfabadi et al., 2011), since the efficiency of protein solubilization are influenced by the chemical composition, structural characteristics and the morphology of microalgae (Ursu et al., 2014). *Chlorella* protein are also proven to be safe for consumption through various clinical and animal studies, and positive health effects such as lower high blood pressure, glucose, and cholesterol levels are seen with the dietary of *Chlorella* protein (Waghmare et al., 2016).

Apart from that, there are several advantages of using microalgae as main source of proteins, for example, rapid growth rate, high productivity, can withstand harsh conditions, ability to be cultivated using exhaust industrial gases and indirectly reducing greenhouse gas emissions (Jeon et al., 2005; Chew et al., 2018a). The cultivation of microalgae does not require freshwater and it can be cultivated using non-arable land that does not affect the need for land to grow food crops. These advantages have attracted huge interests to further investigate the potential of microalgae in pharmaceutical, food, cosmeceutical, and bioenergy applications. Nevertheless, the production cost of biomolecules from microalgae in industrial-scale is relatively high and the commercialization of microalgae technology still remains a challenge to the current industry (Chia et al., 2018). Thus, the development of an efficient technique for scalable production is deemed vital in order to maximize the recovery of biomolecules as well as the industrial profitability.

Conventional separation techniques like membrane separation, column chromatography, precipitation and crystallization often consists of multiple unit operations and requires high consumption of toxic organic solvents. The multiple processing stages consequently contributes to more time consumption and product loss throughout the entire process, and this leads to lower concentrations of the end products. Currently, the industry is searching for alternative techniques to overcome the mentioned drawbacks using more greener and efficient techniques. Hence, three phase partitioning (TPP) was introduced as an efficient approach in extracting and purifying enzymes and biomolecules (Akardere et al., 2010; Gagaoua et al., 2014). TPP is easy to operate, efficient and scalable, where the salt is added to the aqueous solution containing the targeted product, followed by the addition of t-butanol to form three phases. The top phase of TPP is the t-butanol layer, middle phase is the protein precipitate layer and bottom phase is the aqueous layer where the solubility of t-butanol with water will change with the addition of salt. Since it is a three phase system, the targeted product may partition to either of the phases due to the operational conditions and physicochemical properties of targeted product (Avhad et al., 2014).

The partitioning behavior of targeted product depends on the mass transfer phenomenon, where the purification fold and partitioning of end-product can be improved through the increment of mass transfer (Niphadkar and Rathod, 2015). The utilization of ultrasound has been well applied in various processes such as extraction, absorption, bioremediation, and fermentation to improve the mass transfer of targeted products (Gogate and Kabadi, 2009; Sulaiman et al., 2011; Tay et al., 2016). The mass transfer of targeted products is intensified when the ultrasonic waves generate cavitational bubbles in the medium. The shock waves and mechanical shear will be imparted to the surrounding environment due to the collapsing of these bubbles (Avhad et al., 2014). The cavitational phenomenon caused by the difference in mechanical shear and local energy densities will speed up the mass transfer across the phases in the system. Therefore, the integrated approach for protein extraction was conducted to improve the purification and mass transfer of protein.

The aim of the present study was to achieve a maximum recovery of proteins from *Chlorella vulgaris* FSP-E through triphasic partitioning techniques, three phase partitioning (TPP) and ultrasound-assisted three phase partitioning (UATPP). The first attempt was performed using TPP and UATPP with similar operating conditions to extract and purify proteins from microalgae in a single unit operation. Several operating parameters like types of salt, salt saturation, slurry to t-butanol ratio, ultrasonic power, ultrasonic frequency and time, duty cycle and biomass loading were then studied and optimized.

## Materials and Methods

### Chemicals and Reagents

T-butanol, ammonium sulfate ((NH_4_)_2_SO_4_), sodium sulfate (Na_2_SO_4_), magnesium sulfate (MgSO_4_), magnesium acetate (Mg(CH_3_COO)_2_), dipotassium hydrogen phosphate (K_2_HPO_4_), Bradford reagent, and tris-HCl buffer were purchased from R&M chemicals (Malaysia). Bovine serum albumin (BSA) standards were purchased from Merck (Malaysia). All chemicals used were of analytical grade.

### Microalga

The microalga selected is the green microalgae, *C. vulgaris* (strain FSP-E). BG-11 medium was used for pre-culturing the selected microalgae for around 7 days and then the microalgae was cultivated in batch mode for 10–14 days by supplying 5% of CO_2_ continuously. The BG-11 medium used was prepared with: 1.5 g/L of NaNO_3_, 0.03 g/L of K_2_HPO_4_, 0.075 g/L of MgSO_4_.7H_2_O, 0.006 g/L of citric acid, 2 g/L of Na_2_CO_3_, 3.6 g/L of CaCl_2_.2H_2_O, 0.6 g/L of H_8_N_8_CeO_18_, 0.1 g/L of EDTA, 2.86 g/L of H_3_BO_3_, 1.81 g/L of MnCl_2_.4H_2_O, 0.222 g/L of ZnSO_4_.7H_2_O, 0.39 g/L Na_2_MoO_4_.2H_2_O, 0.079 g/L of CuSO_4_.5H_2_O, and 0.049 g/L of Co(NO_3_)_2_.6H_2_O.

The microalgae were cultivated in batch mode using a 1 L indoor glass vessel photo-bioreactor (PBR) by inoculation from the pre-culture cultivated in a PBR (250 mL). Light source was provided continuously to illuminate the culture by mounting external LED lights on the both sides of the PBR. The initial culturing of microalgae and transfer of inoculum from the agar plate to the PBR were performed under a UV laminar flow chamber to prevent biological contamination. The biomass productivity, nitrate content, and pH values of the microalgae culture were monitored during the batch cultivation. The biomass was harvested when the growth of microalgae achieved a stationary phase. The harvested biomass was centrifuged at 6,000 rpm using a centrifuge (Eppendorf, 5430) for 5 min to remove liquid content. The remaining biomass paste was frozen at −20°C for 24 h before freeze-dried prior to the extraction experiments.

### Determination of Protein Content

The proteins extracted were examined and quantified through modified Bradford method (Bradford, 1976). A total amount of sample containing proteins, 0.25 mL, was mixed with 2.5 mL of Bradford reagent and measured at the wavelength of 595 nm using a UV-Vis spectrophotometer. The readings obtained in absorbance unit were converted into protein concentration using the calibration curve performed while using BSA as standard. Calibration curve of proteins concentration was performed using standards in a range of 0–1 mgL^−1^.

### Determination of Separation Efficiency (*E*), Total Protein Content, and Yield (Y)

Separation efficiency of the extracted protein was calculated using Equation (1):

(1) $$\begin{array}{l} E=\frac{V_{M}C_{M}}{V_{T}C_{T}+V_{M}C_{M}+V_{B}C_{B}} \end{array}$$

where V_T_, V_M_, and V_B_ are volume of top, middle and bottom phase, respectively; C_T_, C_M_, and C_B_ are concentration of top, middle and bottom phase, respectively.

The total protein content in microalgae biomass (*P*_*T*_) was calculated using Equation (2) (Safi et al., 2013):

(2) $$\begin{array}{l} P_{T}=N_{ea}\times NTP \end{array}$$

where N_ea_ is the total nitrogen (%) in *C. vulgaris* FSP-E and a value of 4.18% was obtained through elemental analysis; NTP is a constant value of nitrogen-to-protein conversion factor, which is 4.78 (Lourenço et al., 2004; Templeton and Laurens, 2015). Therefore, the final value of *P*_*T*_used in this work is 19.98%. The elemental composition of microalga is carbon %: 49.32, hydrogen %: 8.24, nitrogen %: 4.18 and sulfur %: −0.15.

Yield of proteins (Y) from microalgae biomass was calculated using Equation (3):

(3) $$\begin{array}{l} Y=\frac{V_{M}C_{M}}{\frac{P_{T}}{100}\times W_{i}}\times 100\% \end{array}$$

where W_i_ is the initial weight of biomass microalgae used in the study.

### Three Phase Partitioning (TPP)

The study of TPP was conducted with a total working volume of 10 mL using the predetermined initial conditions. The system was prepared using 5 mL of 30% saturation (NH_4_)_2_SO_4_, 5 mL of pure t-butanol and 0.5 wt% of freeze-dried microalgae biomass. Firstly, the microalgae biomass was dissolved in deionized water before mixing with salt solution. Five milliliters of t-butanol was then added to the mixture of microalgae biomass and salt solution in a small beaker. The mixture was stirred using a magnetic stirrer at 200 rpm for 1 h and was allowed to separate for 30 min at room temperature. Three phases were observed and separated carefully by pipetting them out from the beaker. The intermediate protein precipitate was dissolved in appropriate amount of tris-HCl buffer and analyzed for protein content. The separation efficiency and yield of proteins in all three phases were analyzed and compared with the ultrasound-assisted three phase partitioning process. All the experiments were repeated three times and the mean of the values were reported.

### Ultrasound-Assisted Three Phase Partitioning (UATPP)

Ultrasound-assisted three phase partitioning of proteins was conducted using an ultrasonic water bath. The initial conditions of UATPP such as the working volume, saturation of salt solution, and weight of biomass were similar with TPP for the comparison study. The preparation and mixing procedure of UATPP was similar with TPP, but a glass vial was used for ultrasonic treatment. The initial volume ratio used is 1:1. The glass vial containing the mixture was placed into the ultrasonic water bath, operating with 35 kHz and 100% sonication power for 5 min. The glass vial was then taken out and allowed to separate for 30 min at room temperature. The intermediate protein precipitate was dissolved with tris-HCl and proteins in all three phases were analyzed through the modified Bradford method. All the experiments were done in triplicate and the mean values were reported.

### Optimization of Operating Parameters

The optimization of UATPP was applied for proteins extraction, where the system composed of t-butanol and salt solution with sonication effect. Operating parameters of UATPP which includes the type and saturation of salt, ratio of slurry to t-butanol, sonication power, frequency, irradiation time, duty cycle, and biomass loading, were studied in the optimization step. Initial conditions and subsequent variables for each parameter were shown in Table 1. Types of salt was set as the first parameter to determine the interaction of different salts with t-butanol in order to precipitate out the protein from the biomass. Optimum salt saturation was investigated to reduce the usage of salt in the whole system, where the highest salt saturation studied was up to 60%. The third parameter, slurry to t-butanol ratio was studied before the optimization of sonication properties. The relationship between the volume of slurry and t-butanol used in experiment was studied as it is critical in optimizing the TPP process (Sharma et al., 2000). The sonication parameters were optimized to use energy more efficiently and save operating cost where possible while achieving optimum proteins extraction. The last parameter, biomass loading was examined due to possible change in equilibrium of three phases in the system. The effects of operating parameters were studied using one-factor-at-a-time (OFAT) approach.

**Table 1:** Operating parameters for UATPP.

**No**.	**Operating parameter**	**Initial setting**	**Variables**	**Unit**	**Justification**
1.	Types of salt	(NH_4_)_2_SO_4_	Na_2_SO_4_, MgSO_4_, Mg(CH_3_COO)_2_, K_2_HPO_4_	N/A	First parameter to optimize the salt saturation
2.	Salt saturation	30	20, 40, 50, 60	%	–
3.	Slurry to t-butanol ratio	1:1	1:0.5, 1:1.5, 1:2, 1:2.5	N/A	The ratio of slurry is set as 1 (slurry : t-butanol)
4.	Sonication power	100	20, 40, 60, 80	%	–
5.	Sonication frequency and irradiation time	35 kHz and 10 min	Set A (35 kHz): 2.5, 5, 7.5, 12.5 min Set B (130 kHz): 2.5, 5, 7.5, 10, 12.5 min	kHz, min	–
6.	Duty cycle	100	20, 40, 60, 80	%	100% is continuous sonication
7.	Biomass loading	0.5	0.25, 0.75, 1.00, 1.25	wt%	–

### Field Emission Scanning Electron Microscope FESEM

Field Emission Scanning Electron Microscope (FESEM, Quanta 400F) (FEI, USA) analysis was used to analyze the morphological structure of microalgae biomass before and after extraction (with and without UATPP).

### Statistical Analysis

The data was subjected to one-way ANOVA (with significance *p* ≤ 0.05) data analysis and the mean differences were compared using Tukey HSD *post-hoc* test. All studies were conducted in triplicate and the values were expressed as mean ± standard error. The statistical analysis was performed using IBM SPSS statistics software (SPSS version 26.0 for window; IBM Corporation; United States).

## Results and Discussion

### Comparison Study

The study of TPP and UATPP showed that the addition of sonication resulted in better extraction of proteins from microalgae. The results obtained using both methods were tabulated in Table 2.

**Table 2:** Comparison study between TPP and UATPP.

**Methods**	**Yield (Y, %)**	**Separation efficiency (E, %)**
TPP	25.15 ± 1.04	49.78 ± 0.44
UATPP	40.01 ± 4.51	52.26 ± 2.47

In both cases, the proteins favorably partitioned to the intermediate phase and less proteins partitioned to the top and bottom phase. These precipitated proteins formed in between the top and bottom phases created the three-phase formation (Chew et al., 2018b). Better results were obtained using UATPP compared to TPP due to the mechanism of ultrasonication which has helped to break the cell wall of microalgae. The cavitation bubbles induced by ultrasonic waves disrupts the microalgae cell wall, leading to the release of proteins from microalgae cells. In addition, TPP requires 1 h to partition proteins from microalgae biomass while UATPP only needs 10 min to achieve higher yield of proteins. This significant finding showed that higher yield and better separation efficiency of proteins could be achieved within shorter processing time by UATPP as supported by Pakhale and Bhagwat (2016). The findings are in agreement with Zhang et al.'s work, where the recovery of targeted product increased using UATPP compared to conventional TPP (Zhang et al., 2017). The reduced processing time subsequently leads to lower operating cost via UATPP.

### Optimization of UATPP Process Parameters

#### Types of Salt

**Figure 1:**
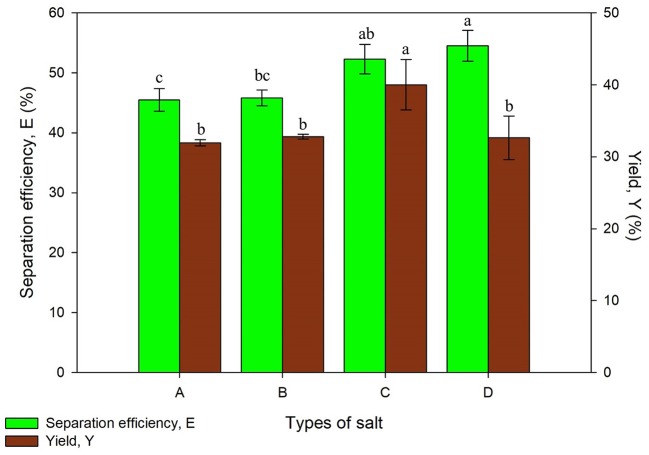
Effect of types of salt on separation efficiency (E) and yield of protein (Y) where A is Na_2_SO_4_, B is MgSO_4_, C is (NH_4_)_2_SO_4_, and D is K_2_HPO_4_. Means with the same letter are not significantly different (One-way ANOVA followed with Tukey's test).

The first parameter that was optimized in this study is the compatibility of t-butanol with various types of salt for protein extraction. Salts type plays an important role in UATPP as the partitioning behavior of proteins is influenced through the interaction of salts and t-butanol depending on the ionic strength of solution. All the systems showed a slight difference (around 9%) in separation efficiency, ranging from 45.48 ± 1.90 to 54.48 ± 2.58%, excluding the system consisting of Mg(CH_3_COO)_2_ from Figure 1. The protein yield for Na_2_SO_4_, MgSO_4_, and K_2_HPO_4_ was comparable, falling in the range of 31.94 ± 0.24 to 32.63 ± 4.04%, while (NH_4_)_2_SO_4_ obtained the highest yield of proteins. Based on the statistical analysis, the yield of protein was significantly affected by the types of salt (*p* < 0.05).

The combination of selected salts with t-butanol have formed a distinct three phase except for Mg(CH_3_COO)_2_, where only two phases were observed with a blur separation interface. This phenomenon may be due to the salting-in effect by Mg(CH_3_COO)_2_ at a particular molality. However, the salting-out effect of Mg(CH_3_COO)_2_ with IL was stronger than K_2_HPO_4_ as stated by Neves et al. (2015), proving that every combination of salt and solvent have to be investigated for their suitability to extract specific products (Neves et al., 2015). (NH_4_)_2_SO_4_ obtained the highest yield of proteins as it has higher hydration capacity and affinity of water compared to other types of salt. The hydration energy of sulfate ions has increased its effective radius by involving large proportions of water molecules and promoting the stability of proteins through hydrophobic interactions. Large ions gather together and isolate the proteins through precipitating the proteins out of the water phase. Besides that, the sulfate ions are positioned in the front part of the Hofmeister series. They interact well with water molecules as they act as dehydrating agent, forming H-bonds as well as dehydrate proteins (Pakhale and Bhagwat, 2016). T-butanol with the presence of high (NH_4_)_2_SO_4_ concentration acts in more lipophilic manner, increasing the hydrophobicity and exclusion from water due to the higher effective dielectric constant of water (Waghmare et al., 2016). Various studies have utilized (NH_4_)_2_SO_4_ in both TPP and UATPP for its salting out ability, and have successfully extracted the desired products (Pakhale and Bhagwat, 2016; Gagaoua et al., 2017). Therefore, the system of (NH_4_)_2_SO_4_ and t-butanol was chosen for further studies.

#### Effect of (NH_4_)_2_SO_4_ Saturation

**Figure 2:**
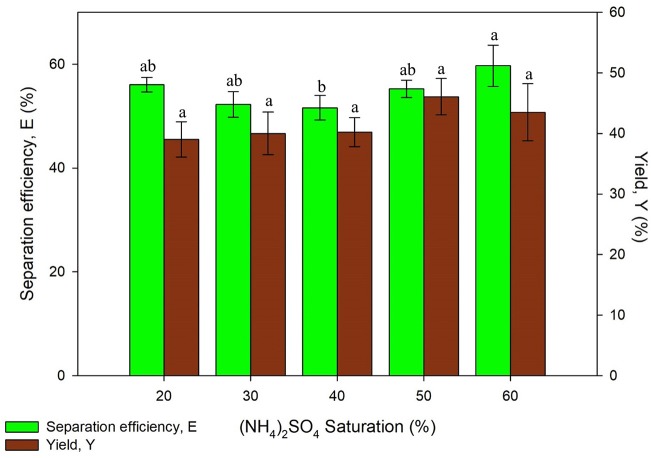
Effect of (NH_4_)_2_SO_4_ saturation on separation efficiency (E) and yield of protein (Y). Means with the same letter are not significantly different (One-way ANOVA followed with Tukey's test).

Saturation of (NH_4_)_2_SO_4_ is a significant parameter on UATPP due to the salting-out phenomenon. The effect of (NH_4_)_2_SO_4_ saturation was investigated from 20 to 60% (w/v). Figure 2 showed that increasing (NH_4_)_2_SO_4_ saturation enhanced the separation efficiency and yield of proteins due to the salting out effect. The protein yield showed an increasing trend along with the increased saturation of salt up to 50% saturation, where it starts to decrease, this is in agreement with results reported by previous research (Niphadkar and Rathod, 2015). The yield of proteins decreased at higher saturation of (NH_4_)_2_SO_4_ (>50%) due to the irreversible denaturation of proteins with too much salt concentration. The maximum separation efficiency was obtained using 60% saturation of (NH_4_)_2_SO_4_ while maximum yield was obtained using 50% saturation of salt. The protein was more likely to be partitioned at higher (NH_4_)_2_SO_4_ saturation which leads to higher separation efficiency due to the stronger salting-out effect. Higher saturation of (NH_4_)_2_SO_4_ will cause water from the solvation layer around proteins to be dissipated more easily. This will lead to the exposure of hydrophobic patches of protein surfaces, causing the interaction with hydrophobic patches of other protein surfaces to occur. As a result, aggregation and precipitation of proteins would be observed in the intermediate phase (Niphadkar and Rathod, 2015). The results obtained by Niphadkar and Rathod (2015) was slightly lower than the result from this experiment, which is 40% (NH_4_)_2_SO_4_. The experiment was conducted under 1:0.5 of crude extract to t-butanol ratio, pH 6, 60% duty cycle with 25 kHz frequency and 3 min irradiation time (Niphadkar and Rathod, 2015). However, it was found that the result obtained was in agreement with the study of Zhang et al. (2017), 50% of (NH_4_)_2_SO_4_ was the optimized (NH_4_)_2_SO_4_ concentration to recover phycocyanin from *Spirulina* (Zhang et al., 2017). The conditions performed for the phycocyanin extraction was 1:1 crude extract to t-butanol ratio, pH7 at 25 kHz frequency with 50% duty cycle. The statistical analysis showed that the yield of protein was significantly affected (*p* < 0.05) by the salt saturation parameter. Lower protein yield was observed in lower (NH_4_)_2_SO_4_ saturation (20%) as it is insufficient to modify the hydrophobic patches surfaces of proteins. Similar results have been observed in the work of Avhad et al. (2014) where lower recovery and purification fold was found at lower (NH_4_)_2_SO_4_ saturation (20% w/v) (Avhad et al., 2014). The highest yield was obtained using 50% saturation of (NH_4_)_2_SO_4_, therefore it was selected for the next study.

#### Effect of Slurry to T-butanol Ratio

The alcohol used for UATPP was set as t-butanol as it is commonly used in past studies (Yadav et al., 2017; Patil and Yadav, 2018). Dhananjay and Mulimani (2009) have stated that at room temperature, t-butanol tends to be kosmotropic, contributing to enzyme-t-butanol co-precipitates that floats above the bottom phase (Dhananjay and Mulimani, 2009). Another unique characteristic of t-butanol is that it could not permeate into folded proteins due to its structure and size, which can avoid protein denaturation (Dennison, 2000). The effect of slurry to t-butanol ratio on UATPP of protein was examined by altering the working volume of t-butanol while the slurry remained constant throughout the experiment. The ratio of slurry to t-butanol such as 1:0.5, 1:1, 1:1.5, 1:2, and 1:2.5, was studied with the optimized (NH_4_)_2_SO_4_ saturation. The working volume of t-butanol was chosen to be adjusted rather than (NH_4_)_2_SO_4_ because previous studies stated that the inherent properties of t-butanol have offered great advantages in partitioned enzyme (Avhad et al., 2014), as it aids in enhancing the protein yield through UATPP.

**Figure 3:**
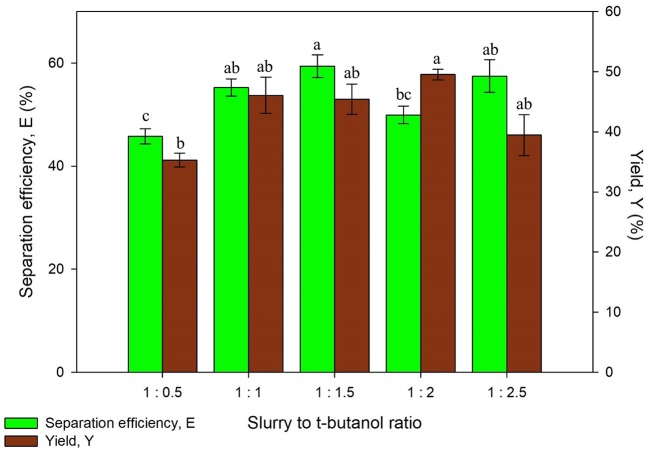
Effect of slurry to t-butanol ratio on separation efficiency (E) and yield of protein (Y). Means with the same letter are not significantly different (One-way ANOVA followed with Tukey's test).

It was observed that the separation efficiency and yield of proteins were rising with increasing slurry to t-butanol ratio from Figure 3. However, both separation efficiency and yield has decreased at the highest ratio (1:2.5). The denaturation of protein was more likely to occur if the volume of t-butanol is high (>1:2), where this was also observed in extraction of enzymes (Narayan et al., 2008). The increment of surface tension caused by high concentration of t-butanol also leads to lower yield and separation efficiency. This is due to the high surface tension that has decreased cavitation (Santos et al., 2008). Kulkarni and Rathod (2014) have reported similar trends where the yield of mangiferin decreased with the increasing ratio of slurry to t-butanol (Kulkarni and Rathod, 2014). The separation efficiency of protein using all systems was more than 50% except from the ratio of 1:0.5, in which the synergistic effect of (NH_4_)_2_SO_4_ to recover proteins could not be achieved since the volume of t-butanol is much less. On the other hand, the yield obtained was varied through different ratios. The protein yield obtained falls in the range of 35.28 ± 1.16–49.50 ± 0.89%, with the highest yield obtained being 1:2 of slurry to t-butanol ratio. Chew et al. (2019) have reported a 1:1 ratio of slurry to t-butanol as the optimum ratio for protein extraction from *Chlorella* sp. which was not in agreement with the obtained result due to different TPP conditions, for example, the optimized salt concentration was 30% in the work by Chew et al. (2019). Thus, the appropriate ratio of slurry to t-butanol should be determined for specific optimized extraction. The ratio of slurry to t-butanol significantly affected the protein yield (*p* < 0.05). The optimum value of separation efficiency and yield were obtained using the ratio of 1:2 and this was selected for next study.

#### Effect of Ultrasonic Power

The ultrasonic power was studied as part of the optimization of sonication in the system. This study was conducted with the optimum values from previous experiments and initial settings of other parameters by varying the power of the ultrasonic bath. The ultrasonic power is an important factor that affects the cost of the process when operated at industrial scale (Niphadkar and Rathod, 2015; Zhang et al., 2017). On top of that, the ultrasonic power has to be optimized to avoid denaturation of proteins. Generation of large amplitude ultrasonic waves may produce more cavitation bubbles through higher ultrasonic power. The higher cavitation effect may result in denaturation of proteins although higher mass transfer can be achieved.

**Figure 4:**
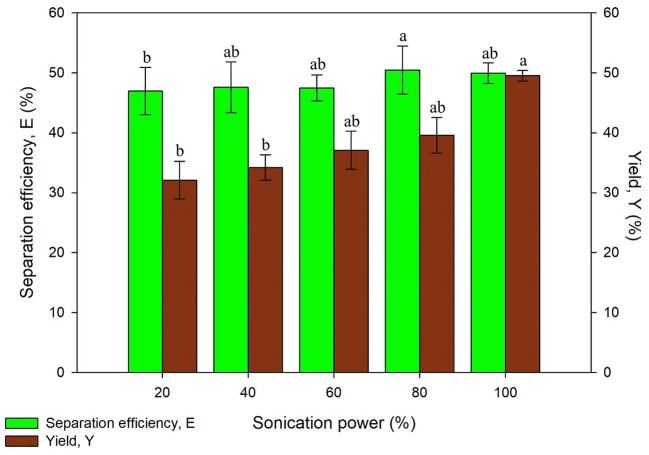
Effect of sonication power on separation efficiency (E) and yield of protein (Y). Means with the same letter are not significantly different (One-way ANOVA followed with Tukey's test).

As illustrated in Figure 4, the results obtained has shown an increasing trend in the separation efficiency and yield of proteins along with higher ultrasonic power. This is because the collapsing of cavitation bubbles has imparted vicious mechanical shear to the system. The mechanical shear induced resulted in the better disruption of microalgae cell wall and subsequently enhanced the mass transfer and protein yield (Zhang et al., 2017). The statistical analysis also showed that the recovery of protein was significantly affected (*p* < 0.05) by the ultrasonic power. The difference of separation efficiency obtained using 20–100% of ultrasonic power did not vary beyond 4%, which was a considerably small difference in values. The separation efficiency obtained indicates that the partitioning behavior of proteins was not influenced much by the ultrasonic power. However, the yield of proteins considerably increased around 10% using 100% of ultrasonic power compared to the lowest yield obtained, which is more favorable in the process. Thus, 100% of ultrasonic power was chosen for further studies due to the highest yield obtained.

#### Effect of Ultrasonic Frequency and Irradiation Time

**Figure 5:**
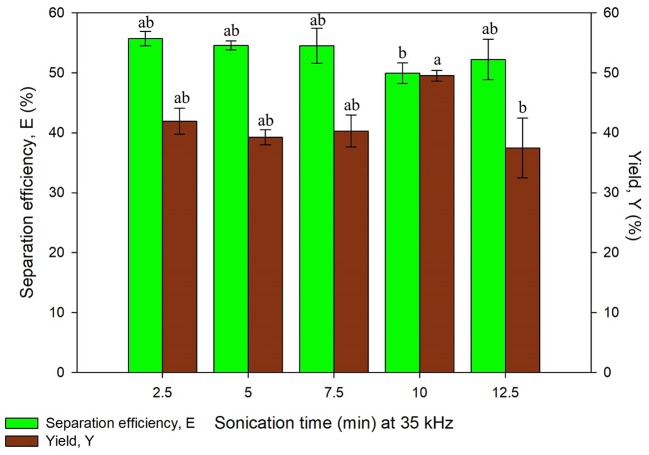
Effect of sonication time at frequency, 35 kHz on separation efficiency (E) and yield of protein (Y). Means with the same letter are not significantly different (One-way ANOVA followed with Tukey's test). Analysis tests were performed with data in Figure 6 (130 kHz frequency).

**Figure 6:**
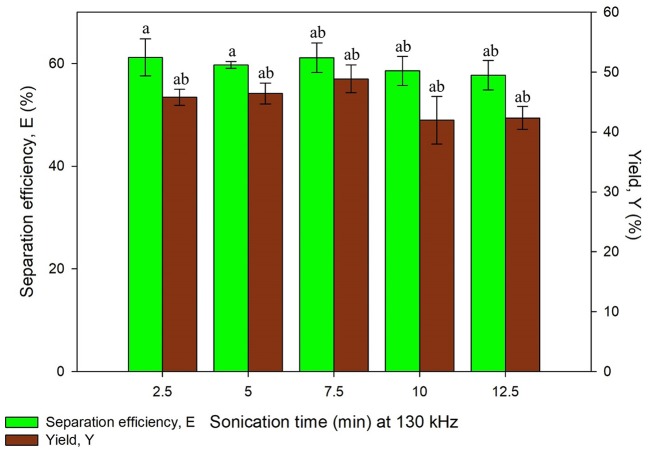
Effect of sonication time at frequency, 130 kHz on separation efficiency (E) and yield of protein (Y). Means with the same letter are not significantly different (One-way ANOVA followed with Tukey's test). Analysis tests were performed with data in Figure 5 (35 kHz frequency).

The study on ultrasonic frequency and time was categorized into two sets, Set A and Set B as listed in Table 1. Set A consists of irradiation time ranging from 2.5 to 12.5 min with the constant frequency of 35 kHz while Set B consisted of same time range with the frequency of 35 and 130 kHz, both the results obtained are illustrated in Figures 5, 6, respectively. Ultrasonic frequency and duration of ultrasonic irradiation was altered as the former regulates power dissipated to the system and the dose of ultrasound was quantified by the latter. The results for yield obtained in 35 kHz shown an increasing trend from 5 to 10 min of irradiation and reduced significantly at 12.5 min.

As for 130 kHz, the yield was in an increasing trend as irradiation time increased and later decreased after 10 min of irradiation time. The reduction of yield at longer irradiation time may be resulted from the excessive ultrasonic irradiation time that altered the protein conformation and led to the degradation of proteins. Furthermore, the increment of temperature from the long irradiation time would contribute to the degradation and activity loss of proteins. The findings were in agreement with previous studies where UATPP of fibrinolytic enzyme and polyphenol oxide were performed (Avhad et al., 2014; Niphadkar and Rathod, 2015). It was observed that the shorter the irradiation period, lower yield was obtained. This is possibly due to insufficient ultrasonic irradiation to disrupt the rigid microalgae cell wall. If the ultrasonic irradiation is not sufficient, the agitation could not lead to the formation of large turbulence in the solvent phase, thus the mass transfer could not be enhanced (Niphadkar and Rathod, 2015). In all sets of experiment, the highest yield was obtained at 10 min of irradiation time for lower frequency, 35 kHz. Higher frequency of ultrasonic has decreased the cavitation yield due to formation of smaller and less energetic cavitation bubbles compared to low frequency, leading to lower yield of products (Kirpalani and McQuinn, 2006). As compared to the study performed by Zhang et al. (2017), the higher frequency (40 kHz) obtained lower purity and recovery of phycocyanin compared to the lower frequency (25 kHz) (Zhang et al., 2017), supporting as well the statement of Kirpalani and McQuinn (2006). The frequency of sonication and irradiation time significantly affected the protein recovery through the statistical analysis (*p* < 0.05). There have been reports that concluded low frequency was favorable for biomolecules extraction (Capote and de Castro, 2007; Kulkarni and Rathod, 2014). Therefore, 10 min of irradiation time with 35 kHz of ultrasonic frequency was selected for the next study.

#### Effect of Duty Cycle

**Figure 7:**
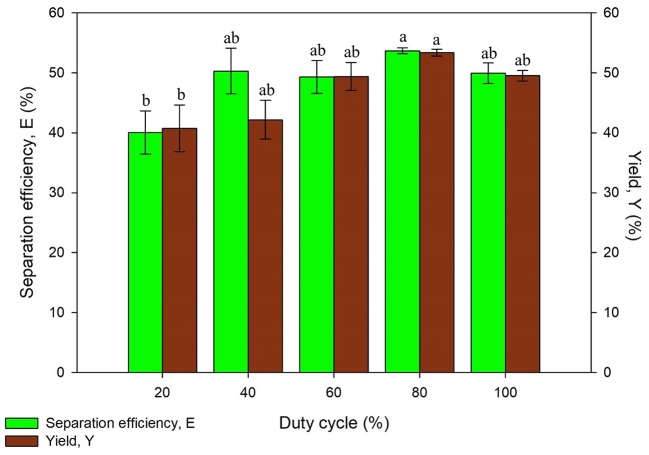
Effect of duty cycle on separation efficiency (E) and yield of protein (Y). Means with the same letter are not significantly different (One-way ANOVA followed with Tukey's test).

Duty cycle was varied by switching the ON and OFF time of ultrasonic process at 35 kHz. Higher percentage of duty cycle has higher fraction of ultrasonic irradiation in one period. It was found that the separation efficiency and yield has increased up to 80% of duty cycle (48 s ON and 12 s OFF) and thereafter it decreases as shown in Figure 7. This is probably a result of the excessive ultrasonic dose of UATPP. The degradation of protein structure could occur at higher duty cycle as the temperature rose along with the mechanical shear caused by the implosion of cavitation bubbles. The results obtained were in agreement with previous studies, where the phycocyanin recovery decreased after 90% of duty cycle (Zhang et al., 2017). However, the purification of serratiopeptidase from *Serratia marcescens* NRRL B 23112 using UATPP only required 40% duty cycle for 5 min to obtain highest purity and recovery (Pakhale and Bhagwat, 2016). The decrement was caused by excessive sonication dose toward the system. Based on the statistical analysis, the duty cycle of sonication significantly affected the protein yield (*p* < 0.05). Sonication can be operated in continuous mode or pulse mode. The pulse mode of sonication to intensify bioprocesses shows great potential due to various advantages. It is preferable as the energy utilized in pulse mode is lower than in continuous mode and more energy efficient as unnecessary heat and energy loss can be avoided. It is stated that the pulse mode of sonication was able to intensify the recovery as well as reduce the occurrence of heat sensitive compounds degradation (Kulkarni and Rathod, 2014). On top of that, the lifespan of transducers can be prolonged in pulse mode compared to continuous mode (Dey and Rathod, 2013). Thus, the duty cycle of 80% was chosen as the optimized condition in this study.

#### Effect of Biomass Loading

Apart from the optimization on sonication, the biomass loading was altered to study its effect on protein extraction via UATPP. The biomass loading in a range of 0.25–1.25 wt% were examined. It is known that the yield of proteins could be increased by increasing the biomass loading. The studies related to aqueous two phase system have proven that protein separation is saturated once the biomass loading has achieved the critical quantity (Selvakumar et al., 2010). However, there is no research reporting the effect of biomass loading on UATPP whereby the equilibrium between the three phases could possibly be affected by the weight of biomass loaded into the system.

**Figure 8:**
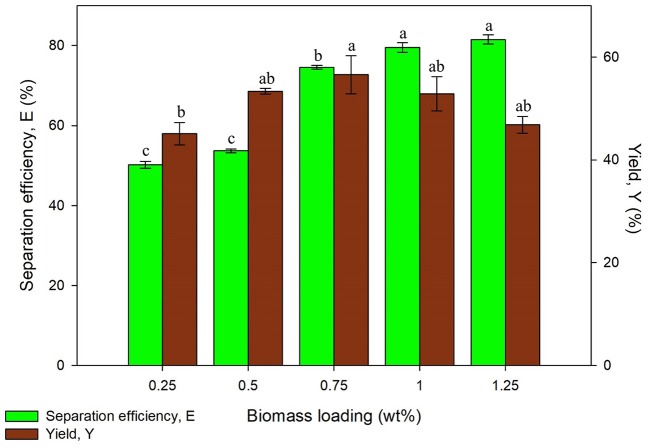
Effect of biomass loading on separation efficiency (E) and yield of protein (Y). Means with the same letter are not significantly different (One-way ANOVA followed with Tukey's test).

From Figure 8, an increasing trend was observed from 0.25 to 0.75 wt% of biomass loading and the protein yield was decreased after 0.75 wt% of biomass. The highest yield was discovered at 0.75 wt% of biomass with moderate separation efficiency. It is the optimum biomass loading because the sonication irradiation was fully utilized to disrupt the microalgae cells, and subsequently improved the protein recovery. Besides, it was observed that the lower protein yield has been obtained by lower biomass loading. This is due to the initial quantity of proteins that already exists in the biomass loading is low, such that the microalgae cell wall were fully disrupted by sonication, and the total amount of proteins present remain the same. It was observed that the proteins recovered at higher biomass loading (>0.75 wt%) was also decreased. The obtained results were found to be in agreement with work of Chew et al. (2019) where the recovery of protein decreased at higher microalgae percentage, 0.75 wt% (Chew et al., 2019). The biomass loaded to the system has influenced the separation efficiency and yield of protein such that higher loading of biomass into the system leads to uneven distribution of ultrasonic irradiation and may cause the incomplete breakage of cell wall for protein extraction. Therefore, a biomass loading of 0.75 wt% was taken for further studies with the consideration of separation efficiency and yield obtained. The statistical analysis showed that biomass loading significantly affected (*p* < 0.05) the yield of proteins in the system.

### Scale Up

The integrated system of sonication and TPP on protein extraction in a larger scale was examined and explored for its feasibility, reliability, as well as the possibility of this technique to be scaled-up. The system was conducted with all the optimized parameters such as 50% of (NH_4_)_2_SO_4_ saturation, 1:2 of slurry to t-butanol ratio, 100% of power, 35 kHz of ultrasonic frequency, 10 min of irradiation time, 80% of duty cycle and 0.75 wt% of biomass loading. The large-scale study was performed with the total working volume of 10 times higher than the lab scale study. A total working volume of 150 mL with 50 mL of slurry and 100 mL of t-butanol were used. The yield obtained in larger scale system (56.57 ± 3.70%) was comparable with the yield obtained with the lab scale system (57.03 ± 1.32%). Relatively high separation efficiency of protein was obtained in large scale as well. Based on the results shown in Table 3, the reliability of this technique in scale-up has been verified and could be further up-scaled toward an industrial scale.

**Table 3:** Comparative evaluation of other studies using TPP and ultrasound-assisted TPP.

**Studies**	**Target compound**	**Findings**	**References**
Extraction of protein from *Chlorella* sp. in lab-scale	Protein	A total yield of 56.57% ± 3.70 and 74.59% ± 0.45 separation efficiency were obtained	This study
Extraction of protein from *Chlorella* sp. in large-scale	Protein	A total yield of 57.03% ± 1.32 and 70.88% ± 1.02 separation efficiency were obtained	This study
Recovery of astaxanthin from *Paracoccus* NBRC 101723 using ultrasound-assisted three phase partitioning (UA-TPP)	Astaxanthin	Recovery of 428 μg/g of wet biomass was achieved using 40% (NH_4_)_2_SO_4_, 1:0.75 biomass to t-butanol ratio assisted with sonication as pretreatment using 100% amplitude for 20 s	Chougle et al., 2014
Ultrasound-assisted three-phase partitioning of polyphenol oxidase from potato peel (*Solanum tuberosum*)	Polyphenol oxidase	70% recovery of polyphenol oxidase and 6.3 purification factor were obtained using 40% (NH_4_)_2_SO_4_, 1:1 extract to t-butanol ratio, pH 7, 40% duty cycle with 25 kHz frequency	Niphadkar and Rathod, 2015
Concentration and characterization of microalgae proteins from *Chlorella pyrenoidosa* (three phase partitioning)	Protein	78.1% w/w protein concentration was obtained using 40% (NH_4_)_2_SO_4_, pH 6, 1:1.5 of slurry to t-butanol ratio with enzymatic treatment (combination of Stargen and Carezyme)	Waghmare et al., 2016
Ultrasound-assisted three phase partitioning of phycocyanin from *Spirulina platensis*	Phycocyanin	94.3% recovery of phycocyanin with 6.69 purification factor were obtained using 50% (NH_4_)_2_SO_4_, 1:1 crude extract to t-butanol ratio, pH 7, 50% duty cycle with 25 kHz frequency	Zhang et al., 2017

A comparative study was performed using the results obtained in current study and literature from previous studies. There are three studies that found that 40% (NH_4_)_2_SO_4_ was the suitable salt saturation % for recovering their targeted compounds (Chougle et al., 2014; Niphadkar and Rathod, 2015; Waghmare et al., 2016), which was not in agreement with our findings. It was found that most of the condition utilized in the mentioned studies was not similar with the current study and the recovery of compounds obtained was generally higher. The recovery of protein in Waghmare et al. (2016) was performed with enzymatic treatment which requires higher slurry to t-butanol ratio compared to this study in order to achieve optimum results. The results obtained in the study of Zhang et al. (2017) was similar with results obtained in current study, which requires 50% (NH_4_)_2_SO_4_ and low irradiation frequency (25 kHz) to recover phycocyanin from microalgae, a different type of protein.

### Field Emission Scanning Electron Microscopy (FESEM) Characteristics of Microalgae Surfaces

**Figure 9:**
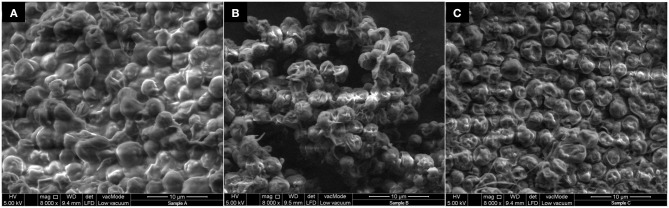
FESEM images of microalgae cells **(A)** before sonication treatment; **(B)** after ultrasound with water as medium; **(C)** after ultrasound with UATPP medium.

Figure 9 shows the FESEM images of the surface of microalgae before sonication treatment, after sonication treatment (with water as medium) and after UATPP treatment (with solvents of UATPP). In Figure 9A, the surfaces of microalgae before any sonication treatment were observed to be smooth and round-shape cells that are clumped on the surface. After treatment with sonication, the microalgae cells shrunk and became rougher compared to the microalgae cells without ultrasonic treatment (Figure 9B). However, the structure of microalgae was more severely altered, and became flat and more shrunken after the treatment with UATPP (Figure 9C). This indicates that UATPP can deal more disruptive damage to the microalgae cell wall compared to treatment with sonication only, where the use of TPP solvents can further help to recover higher portions of the compounds in microalgae. The obtained results have proven that UATPP is very effective in disrupting the microalgae cells and leads to the enhancement of protein extraction.

## Conclusions

In this work, proteins from *C. vulgaris* FSP-E was successfully extracted using sustainable bioprocessing technique, ultrasound-assisted three phase partitioning. The enhanced yield and separation efficiency with reduced processing time and capability to be scaled up, indicates that UATPP is an effective approach for future integrated bio-separation technique for biomolecules extraction from microalgae. The optimized conditions for protein recovery was identified and discussed to move towards the realization of producing low cost nutritional food for the society as well as to develop and improve the current downstream bioprocessing techniques.

## Data Availability Statement

All datasets analyzed for this study are included in the manuscript.

## Author Contributions

SC contributed to the writing of the manuscript, especially the original draft, methodology and the formal analysis of study. KC reviewed and edited the draft prior to the submission. PS contributed to the funding acquisition and supervised the project. HZ assisted in project administration and funding acquisition. D-TC was involved in the investigation of the study. YT contributed into funding acquisition of the research.

### Conflict of Interest

The authors declare that the research was conducted in the absence of any commercial or financial relationships that could be construed as a potential conflict of interest.
